# Evaluation of hair cortisol as an indicator of long-term stress responses in dogs in an animal shelter and after subsequent adoption

**DOI:** 10.1038/s41598-022-09140-w

**Published:** 2022-04-21

**Authors:** Janneke Elisabeth van der Laan, Claudia Maureen Vinke, Saskia Stefanie Arndt

**Affiliations:** Animal Behaviour Group, Department of Population Health Sciences – Division of Animals in Science and Society, Faculty of Veterinary Medicine, Utrecht University, P.O. Box 80166, 3584 CM Utrecht, The Netherlands

**Keywords:** Behavioural ecology, Animal physiology, Endocrinology

## Abstract

Shelter dogs are exposed to a variety of stressors. Among non-invasive techniques, hair cortisol concentration (HCC) is suggested an easy to collect biomarker for giving insight into long-term stress responses. We evaluated HCC as an indicator of long-term cortisol responses in dogs in an animal shelter over different chronological time points during sheltering and after adoption. Hair samples were collected from the neck region following a shave/re-shave protocol of shelter dogs (total n = 52) at four different time periods: T1 intake at shelter (pre-shelter period, n = 51); T2 after 6 weeks in the shelter (n = 23); T3 6 weeks after adoption (n = 24); T4 6 months after adoption (n = 22). HCC at T2 was significantly higher than HCC at T1, T3 and T4 (effect of sample collection moment: F_3,41_ = 12.78, *p* < 0.0001). The dog’s weight class, age class, sex, reason for admission, kennel history and melanin type also explained HCC variability. No significant difference in HCC was found between shelter dogs T1 and control pet dogs in their own homes (n = 20, one sample, t = − 1.24, *p* = 0.219). A significant but moderate positive correlation between HCC and urinary cortisol:creatinine ratios was found (т = 0.3, *p* < 0.001). As HCC increased in the shelter, the use of this non-invasive parameter appears a useful additional tool in dog welfare research.

## Introduction

Dogs can suffer from chronic stress in environments that are sub-optimal to their needs, such as in many kennel environments^1–3^. Chronic stress may exceed the animals’ adaptive capacity and thus, threaten its welfare state. Chronic stress may even elicit medical and behavioural problems in the long term^4^. Therefore, to improve canine welfare, reliable and feasible non-invasive indicators of long-term stress levels need to be evaluated.

One indicator of the stress response frequently used in animal welfare studies is the reactivity of the hypothalamic–pituitary–adrenal (HPA) axis, the primary stress-responsive physiological system^5^. The most reliable and widely used biomarker of HPA axis activity is considered to be cortisol, the primary glucocorticoid of dogs and many other species^6^. Cortisol levels in blood, saliva, urine (urinary cortisol:creatinine ratio, UCCR), or feces have long been evaluated in stress research in many species^5^. However, such cortisol matrices only provide information about relatively short time periods, i.e., minutes to hours, and can therefore be influenced by short-term stressors such as sample collection and circadian patterns^7^. In addition, certain sampling methods are not always applicable in practice and invasive procedures such as blood sampling should generally be avoided in non-lab settings. Therefore, these traditional measures are often considered not feasible and inefficient to evaluate chronic stress, which emphasises the necessity of finding alternative and preferably non-invasive measures. Hair cortisol concentration (HCC), for instance, appears an important novel biological matrix to explore, to quantify long-term cortisol concentrations in both humans and animals^8,9^. Although the mechanisms of cortisol deposition in hair is not yet fully understood^10^ (e.g., through the blood during growth or local cortisol synthesis in the hair follicle), hair cortisol concentration (HCC) in humans and animals is affected in association with adversity^11^ and prolonged exposure to stressors^12^ and is suggested as a new tool in clinical practice^13^. HCC is known to be stable for more than one year^14,15^, which highlights its practical advantage. Therefore, HCC is considered a feasible retrospective biomarker to evaluate exposure to long-term stressors, but needs more validation for different species.

### HCC in dogs

For dogs, collecting a single sample of hair instead of multiple samples of saliva, urine or feces is feasible for practitioners, veterinarians, and researchers interested in long-term cortisol levels, as this method is non-invasive, and the sample is easy to collect and store at any time. In dogs, HCC has been found to correlate with fecal^16^ and salivary cortisol levels^17^. However, Bryan et al.^18^ did not find significant correlations between cortisol in hair and feces or saliva of dogs in kennels, but cortisol immunoreactivity was less variable in hair than in saliva or feces, supporting the hypothesis that data from hair samples reflect baseline cortisol levels in dogs.

HCC in dogs has been measured in studies monitoring responses to social or environmental stressors. For instance, Siniscalchi et al.^19^ suggested that HCC appears to reflect the dog’s chronic state of emotional reactivity as dogs’ HCC was associated with behavioural responses to different acoustic stimuli. Also, HCC of dogs was negatively associated with the number of so called socialization practices employed in commercial breeding dogs, such as the number of exposures to visitors or new objects^20^, and positively correlated with the time that dogs were regularly left alone (average time: 3.7 h/day)^21^. HCC was also higher in caged dogs on dog meat farms than in pet dogs^22^. In addition, HCC was lower in dogs who experienced positive human interactions, but higher in dogs that showed stranger-directed aggression^23^, suggesting that negative experiences, including lower socialization practices, are associated with higher HCC levels. However, more variable and complex responses to different types of stressors can occur. In Border Collies, higher HCC was associated with questionnaire reported psychosocial stressors and engaging in flyball competitions, but lower HCC was associated with increased anxiety and epilepsy^24^. Interestingly, HCC of owners and dogs were correlated^25^, and HCC related to the owner-dog relationship and owners’ personality^26^. The latter two studies found no effect of physical activity on HCC, which is contrary to expectations as activity is known to increase cortisol levels.

The deposition of cortisol in the hair can be influenced by factors such as hair colour and season of sampling, highlighting the need to include these factors in analyses. For example, Bennet and Hayssen^17^ observed a difference in HCC of different hair colours from the same dogs in German Shepherds and Labradors, where black (eumelanin) hairs had lower levels of HCC than yellow (pheomelanin) hairs and agouti hairs had intermediate HCC. Similarly, Bowland et al.^27^ found light dog fur (yellow, red, or white) to show higher HCC than dark (or sable) fur. However, other studies did not find any differences in HCC between hair colours of dogs^21,28^, but this might be due to different classifications or grouping of hair colours such as black vs non-black, light vs dark or melanin type. Furthermore, season of collection can influence HCC in dogs, with levels being higher in winter compared to summer^23,25^. Interestingly, sex seems to influence HCC in dogs, with females having higher HCC levels^25–27^.

However, although already increasingly used in stress and welfare studies as described above, HCC in dogs has not yet been validated thus far as a measure of long-term HPA-axis reactivity. Therefore, additional studies evaluating HCC in different circumstances are needed and collection methods must be more precise and standardized due to the variable factors of influence.

### HCC as a measure of long-term HPA-axis reactivity in shelter dogs

This study aimed to further validate HCC to measure chronic stress in dogs. For research in shelters, HCC can be an interesting measure with multiple practical applications, e.g., evaluating retrospective HCC levels at intake^6^ and subsequent HCC monitoring with shave/re-shave methods^10,29^ during the shelter period to provide more specific insight in longer-term stress levels over the different chronological phases of sheltering and after adoption into a new home. Therefore, in this study we aimed more specifically to (1) compare HCC in shelter dogs at the moments of relinquishment (pre-shelter), during the shelter period (in-shelter) and after adoption (post-shelter) and compare pre-shelter levels with those of control pet dogs; and (2) explore the relationship between HCC and urinary cortisol levels (UCCR) of the same dogs at the shelter and after adoption.

In an earlier study, urinary cortisol responses in dogs in the shelter were higher than after adoption^30^. Likewise, for this study in-shelter HCC levels were also expected to be higher than post-shelter HCC levels, and higher than HCC of pet dogs. Pre-shelter HCC levels were expected to be more variable, as dogs could come from all kinds of situations before entering the shelter, including high and low stressful situations. HCC levels 6 weeks in-shelter and 6 weeks post-adoption were expected to correlate with urinary cortisol levels of urine samples collected during in-shelter and post-adoption, similar to the correlations of cortisol levels with other matrices in the literature as described above.

## Materials and methods

### Subjects, demographics, and housing

Fifty-two shelter dogs admitted to the largest animal shelter in the Netherlands (Animal Shelter DOA) between October 2018 and August 2019 were included (for demographics, see Supplementary Table 1). Exclusion criteria for the dogs to participate in the study are described earlier in more detail^30^, and comprised being affected by a physical health condition, high levels of anxiety- or aggression-related behaviour, being younger than 1 or older than 13 years of age, and being housed in pairs. The final shelter dog group had a mean age of 3.8 years (range 1–13 years), with 18 females (6 neutered, 9 entire, 3 unknown) and 34 males (12 neutered, 22 entire). The duration of stay in the shelter for these dogs ranged from 5 days up to 8 months, but most stayed in the shelter for at least two weeks. Based on the appearance, an experienced shelter employee assigned the dogs to a breed group^31^. Breed labelling of shelter dogs is highly unreliable^32,33^, but we labelled the dogs in order to match a control group. Dogs were housed as described earlier^30^: individually, in kennels with a glass-fronted indoor and bar-fronted outdoor enclosure, and kennels were only accessible to staff, volunteers, and the researchers of this study.

A control group of twenty pet dogs, balanced for characteristics of the shelter dog group on breed group^31^, size (body weight class), age class^30^, and sex (Supplementary Table 1), housed in their regular home environment, was recruited via social media and dog professionals. The pet dog group had a mean age of 3.6 years (range 1–11 years), with 8 females (7 neutered, 1 entire) and 12 males (10 neutered, 2 entire). Pet dogs were housed as usual for them, at home with their owners, following their normal daily routine which differed per owner, as altering routine could be a stressor for dogs.

### Sample collection moments

**Figure 1:**
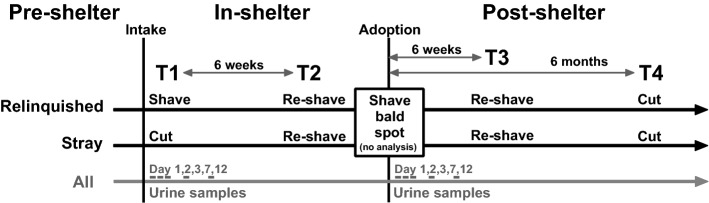
Timeline of shelter dog hair and urine sample collection moments: after intake (T1), after 6 weeks in the shelter (T2), 6 weeks after adoption (T3) and 6 months after adoption (T4). Stray dogs were cut instead of shaved at intake to prevent that a large bald spot was visible if dogs would be recollected by their owners.

Hair samples of shelter dogs were collected via a shave/re-shave protocol, where the same spot was resampled, at several time periods in- and post-shelter with equal regrowth periods in between to assure coherent time periods per sample (see Fig. 1): after intake (T1), after 6 weeks in the shelter (T2), 6 weeks after adoption (T3) and 6 months after adoption (T4, cut by new owners). Participating new owners of the shelter dogs received written instructions and tools for urine collection and hair sampling at T4 sample collection period. Hair samples of control pet dogs were collected once.

### Collection hair samples

Hair samples of dogs were preferably shaved to the level of the skin to remove all hair from the sample area, except for being cut with scissors when the dog was scared for the noise of the razor or when the sample was taken by the owners 6 months after adoption. Also, when dogs entered as a stray, a smaller intake sample was cut for aesthetic reasons, as stray dogs can be recollected by their owners within two weeks. Our pilot study with pet dogs (n = 5) revealed no differences in HCC in shaved versus cut hair samples (see Supplementary Fig. 1, two-tailed paired-samples t-test, t = − 0.57, *p* = 0.597), therefore both shaved and cut samples were included in the analysis. In total, exactly half of the samples were shaved, and half were cut. Not all hair samples for all shelter dogs were collected, due to recollection of the dogs from the shelter within 6 weeks or no participation after adoption.

Where possible, a shave/re-shave protocol was used^16,18^. A 3 × 3 cm area was shaved or cut bald on T1 and pre-adoption to allow a re-shave of newly grown hair at 6 weeks in-shelter and 6 weeks post-adoption. Hairs were collected following a standard protocol in the dorsal neck region as this region is often used in other studies and is easy to access^23–25,28^. The researcher or owner wore nitrile gloves and shaved (Wahl® professional Cordless Trimmer—Super Trim, Type 1592) or cut (blunted scissors) the dog’s hair as close to the skin as possible. Razor and scissors were cleaned between every sample collection. Hairs were kept in aluminium foil in an envelope in a dark closet until analysis, which occurred 6 – 18 months after sampling.

### Hair selection, processing, and analysis

Both wool and guard hair were used for analysis, as these are strongly correlated^23^. From samples T2 and T3, longer and therefore older hairs were removed to prevent contamination of hair outside of the shave/re-shave area. In the case of small amounts of differently coloured hairs being present in the samples, these hairs were removed to make all the samples from the same dog unicoloured to control for colour. The predominant hair melanin type was visually registered^17^. Shelter dog samples per dog were predominantly eumelanin (n = 24) or pheomelanin (n = 13), with fewer white (n = 5), agouti (n = 1) or mixed (n = 9) samples. Pet dog hair samples were eumelanin (n = 11), pheomelanin (n = 2), white (n = 6) and mixed (n = 4).

The hair processing protocol was adapted from previous studies^12^. Samples were washed twice with isopropanol and dried in a stove at 37 °C. Hair samples were cut into small pieces and powdered using metal beads (Lab Services BV Biospec beads, 3.2 mm) in a TissueLyser II (QIAGEN). 1.5 mL methanol was added to each powdered hair sample (30 ± 5 mg, samples that weighed less were marked) for steroid extraction. Samples were incubated overnight at room temperature on an end-over-end roller. 1 mL of the methanol supernatants were dried in a Speed Vac Concentrator (CentriVap Concentrator Labconco, 42 °C). Supernatants were dissolved in phosphate buffer provided by the commercial ELISA essay kit (Salimetrics, LLC), with diluent amount differing per hair sample weight: < 10 mg sample in 55 µL diluent, 10–14 mg in 65 µL, 15–24 mg in 135 µL, > 25 mg in 200 µL.

Cortisol determination with the ELISA kit was performed according to the protocol provided by the manufacturer. Four microtitre plates were used for the ELISA, with samples of one dog assigned to one plate to prevent inter-assay differences within one dog. Plates were read at optical density 450 and 490 nm. Results were expressed as cortisol concentration in pg of cortisol per mg of hair sample. Samples were run in duplicate and inter-assay coefficient of variability ranged from 3.1% to 16.5%; intra-assay variability ranged from 1.3 to 3.1 µL/dL.

### Urinary cortisol:creatinine ratio

For shelter dogs, urine samples were collected on day 1, 2, 3, 7 and 12 both during their time in the shelter and after adoption. Samples were collected after intake in the shelter (by the researchers) and after adoption (by the new owners). Urine was collected between 6:00–12:00 AM (median 8:36 AM). Naturally voided morning urine was captured mid-stream with a ladle and transferred with a pipette to a polyproetheen tube. If the dogs in the shelter were not naturally urinating outside of their kennel, urine on the kennel floor was used. As no significant difference was found in urinary cortisol:creatinine ratio (UCCR) between urine samples of the same dog on the same morning captured directly versus collected from the kennel floor (n = 19, Paired Wilcoxon test: V = 117.5, *p* = 0.376), samples from both methods were included for statistical analysis.

Samples were stored and analysed by the University Veterinary Diagnostic Laboratory of the Faculty of Veterinary Medicine at the Utrecht University, the Netherlands, as described earlier^30^.

### Data handling and statistics

Data were stored and cleaned in Microsoft Excel® files (Microsoft Corporation). Statistical software program RStudio (version 1.0.136 – ©RStudio, Inc.) was used for linear mixed model analysis with the package ‘Nlme’^34^ and Spearman’s rank correlation with the package ‘Hmisc’. Graphs were created in Graphpad Prism (version 8.3.0 ©GraphPad Software, LLC).

Three HCC outliers (mean ± 3s.d. per time point T) were removed: one on T1, T2 and T4. HCC data was right-skewed and therefore (natural) log transformed before statistical testing and back transformed for interpretation. Back transformed (exp) log model values resulted in ratios, with a ratio < 1 meaning a lower value and > 1 a higher value than the reference mean. Alpha level was set at *p* < 0.05. For mixed models, 95% confidence intervals (CI) ranges < 1 or > 1 were considered significant.

UCCR mean of day 1 + 2 + 3 + 7 + 12 per dog was calculated to determine one comprehensive outcome for both in-shelter and post-adoption. The relation between UCCR means and corresponding original HCC outcome on T2 and T3 was evaluated with a Spearman’s rank correlation test for non-parametric variables, as both UCCR and original HCC were not normally distributed (Shapiro–Wilk tests and visual inspection of boxplots and quantile–quantile plots).

HCC data of I) T1 of shelter and pet dogs and II) T1 of relinquished versus stray shelter dogs were compared using a two-tailed t-test on the log transformed HCC data.

A linear mixed effects model was fit on the outcome variable HCC data of the shelter dogs with a fixed effect for ‘sample collection moment’ (T1-4) and a random effect for ‘dog ID’ (individual identity). A full model with variables described below was fitted and variables were dropped with the ‘drop1’ function based on a backward selection approach, using the Akaike information criterion (AIC) to determine the best model fit with Maximum Likelihood estimation. The best fitting model included the factors as seen in Table 1 and described in the results. Variables dropped from the model based on AIC were: ‘sample collection moment*body weight class’, ‘season of collection’ (Dec-Feb/March–May/June-Aug/Sept-Nov), ‘sample collection moment*kennel history’ (no/unknown/yes) and ‘neuter status’ (no/unknown/yes). To test for the best fitting model using the AIC, various correlational and variance structures were added to the model. The best fit was a model with a correlation structure (continuous autoregressive model of the order 1, CAR1) and variance transformation (weights = varPower for ‘sex’). Restricted Maximum Likelihood estimation was used for the final model. Models were evaluated by visual inspection of the residuals (normality and constant variance).

**Table 1:** Model results of hair cortisol concentration in the shelter dog group.

			Intervals		Conditional F-test			
			EP	95% CI	F	NumDF	DenDF	Sign
Reference category	T1, 1–4 year, male, < 10 kg, relinquished by owner, no kennel history, eumelanin type, intact (not neutered)		13.96^1^	10.94–17.81	10,111.65	1	42	< .0001
Sample collection moment	T2 versus T1		1.36^2^	1.12–1.65	12.78	3	41	< .0001
	T3 versus T1		1.22^2^	1.00–1.50				
	T4 versus T1		1.27^2^	0.99–1.64				
Weight class	10–20 kg versus < 10 kg		0.85^3^	0.72–1.02	13.82	3	42	< .0001
	20–30 kg versus < 10 kg		0.76^3^	0.63–0.93				
	> 30 kg versus < 10 kg		0.65^3^	0.54–0.77				
Sex	Female versus male		1.27^4^	1.11–1.45	10.76	1	42	0.0021
Relinquishment reason	Stray versus relinquished		0.69^5^	0.59–0.81	4.73	2	42	0.0140
	Crisis boarding versus relinquished		0.89^5^	0.60–1.32				
Kennel history	Had kennel history versus no kennel history		0.90^6^	0.70–1.15	4.18	2	42	0.0221
	Unknown versus no kennel history		1.46^6^	1.18–1.81				
Sample collection moment * Melatonin type	Pheomelanin versus eumelanin	T1	1.07^7^	0.87–1.31	2.73	16	41	0.0049
		T2	0.63^7^	0.42–0.93				
		T3	1.00^7^	0.73–1.37				
		T4	1.18^7^	0.85–1.64				
	White versus eumelanin	T1	1.25^7^	0.93–1.67				
		T2	0.98^7^	0.65–1.48				
		T3	2.03^7^	1.37–3.02				
		T4	1.38^7^	0.85–2.23				
	Agouti versus eumelanin	T1	0.96^7^	0.57–1.63				
		T2	2.05^7^	1.20–3.50				
		T3	0.88^7^	0.51–1.51				
		T4	1.25^7^	0.72–2.18				
	Mixed versus eumelanin	T1	0.95^7^	0.74–1.21				
		T2	0.71^7^	0.50–1.02				
		T3	0.78^7^	0.53–1.15				
		T4	0.68^7^	0.49–0.95				
Sample collection moment * Age class	5–7 year versus 1–4 year	T1	0.80^8^	0.63–1.01	6.44	8	41	< .0001
		T2	1.76^8^	1.24–2.51				
		T3	0.52^8^	0.34–0.80				
		T3	0.32^8^	0.19–0.55				
	8–13 year versus 1–4 year	T1	1.26^8^	0.95–1.67				
		T2	1.12^8^	0.78–1.60				
		T3	1.22^8^	0.85–1.74				
		T4	0.97^8^	0.64–1.48				

Estimated parameter (EP) and 95% confidence intervals (CI) of HCC at different sampling moments and other factors in the model which significantly explained HCC variability. Conditional F-testing revealed F, DFs and significance of the different terms in the model.

^1^Estimated mean in reference age class, sex, weight class, relinquishment reason, kennel history, melatonin type, neuter status and sample collection moment T1.

^2^Estimated ratio of mean of specified sample collection moment and mean at T1.

^3^Estimated ratio of mean of specified weight class and mean in reference weight class.

^4^Estimated ratio of mean of specified sex and mean in reference sex.

^5^Estimated ratio of mean of specified relinquishment reason and mean in reference relinquishment reason.

^6^Estimated ratio of mean of specified kennel history information and mean in reference kennel history information.

^7^Estimated ratio of mean of specified melatonin type and mean in reference melatonin type at same sample collection moment.

^8^Estimated ratio of mean of specified age class and mean in reference age class at same sample collection moment.

### Ethical note

Methods in this study were performed in accordance with relevant guidelines and regulations. All owners agreed and volunteered to participate in this study and signed informed consent. All experimental protocols were approved by the institutional committee Utrecht Animal Welfare Body of Utrecht University, The Netherlands. The study was carried out in compliance with the ARRIVE guidelines.

## Results

### Comparison shelter dog intake samples with pet dog samples

**Figure 2:**
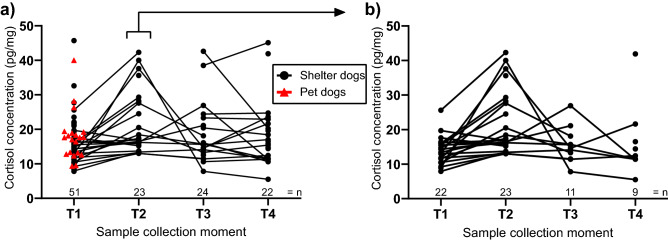
Hair cortisol concentration (pg/mg, original values) at different sample collection moments (T1-4). For (**a**) shelter dogs (“bullet”, total n = 52) and pet dogs (“red triangle”, n = 20), and (**b**) selection of the shelter dogs with an available sample on T2 (6 weeks in-shelter, n = 23), this is only the subset of dogs that were not yet adopted at T2, where the other dogs were not in the shelter anymore. N.B. Individual responses are connected with lines but do not imply continuous sampling.

No significant difference in HCC was found between shelter dog intake samples (T1, mean ± s.d.: 16.0 ± 6.8 pg/mg) and control group samples of pet dogs (17.9 ± 7.1 pg/mg; two-tailed t-test, sample estimated mean difference [ratio] = 1.13, 95% CI = 0.73–1.08, t[69] =  − 1.24, *p* = 0.219, Fig. 2A).

### HCC in shelter dogs

In the shelter dog group, the factors ‘sample collection moment’, ‘weight class’, ‘sex’, ‘reason for admission to the shelter’ and ‘kennel history’ and an interaction effect between ‘sample collection moment’ and ‘age class’ and between ‘sample collection moment’ and ‘melanin type’, significantly explained HCC variability. See Table 1 for full mixed model results with estimates in ratio, 95% confidence interval and F-test statistics of sample collection moment effect, other main effects, and interaction effects as described below).

#### Sample collection moment

Regarding the moment of sampling, the 6 weeks in-shelter HCCs (T2, mean ± s.d.: 21.8 ± 9.4 pg/mg, n = 23) were significantly higher than the intake sample of the shelter dogs (T1, 16.0 ± 6.8 pg/mg, n = 51), where post-adoption samples were not (T3, 17.7 ± 8.5 pg/mg, n = 24 and T4, 18.4 ± 9.5 pg/mg, n = 22, see Fig. 2a for the data of all dogs, 2b for the subset of dogs that were not adopted yet at T2 and therefore had an available sample at T2).

#### Other main effects

Overall, low weight dogs (i.e., smaller dogs, < 10 kg, 20.2 ± 7.9 pg/mg, n = 18 or 35% of all dogs) had higher HCCs than larger weight dogs (20–30 kg, 17.4 ± 10.4 pg/mg, n = 11 or 21%; and > 30 kg, 13.3 ± 3.4 pg/mg, n = 11 or 21%). Female dogs (19.8 ± 9.2 pg/mg, n = 18) had higher HCCs than male dogs (16.9 ± 7.8 pg/mg, n = 34). Relinquished dogs (18.6 ± 8.0 pg/mg, n = 33) had higher HCCs than stray dogs (15.3 ± 7.6 pg/mg, n = 16), but a t-test revealed no significant difference between HCCs of relinquished versus stray dogs at shelter intake (T1, relinquished 15.6 ± 5.0 pg/mg versus stray 13.8 ± 4.4 pg/mg, two-tailed t-test, sample estimated mean difference [ratio] = 1.13, 95% CI = 0.92–1.38, t^46^ = 1.22, *p* = 0.229). Dogs with an unknown kennel history (18.5 ± 9.3 pg/mg, n = 37) had higher HCCs than dogs that had not stayed in a kennel environment before (16.0 ± 5.2 pg/mg, n = 8).

#### Interaction effects

The sample size per sample collection moment for the interaction effects was often very low, therefore the following results need to be interpreted with caution and only sample size is mentioned. A significant interaction effect between ‘sample collection moment’ and ‘age class’ was found, where dogs from age class 5–7 years had lower HCC on T3 (n = 2) and T4 (n = 1) but higher HCC on T2 (n = 4) compared to dogs from age class 1–4 years (1–4 years: T2 n = 15; T3 n = 18; T4 n = 18). Another significant interaction effect was found between ‘sample collection moment’ and ‘melanin type’, with eumelanin samples having higher HCC than pheomelanin samples on T2 (n = 14 versus n = 2), lower HCC than agouti on T2 (n = 14 versus n = 1), lower HCC than white samples on T3 (n = 13 versus n = 3), and higher HCC than mixed samples on T4 (n = 8 versus n = 6).

### Relation HCC with urinary cortisol/creatinine

**Figure 3:**
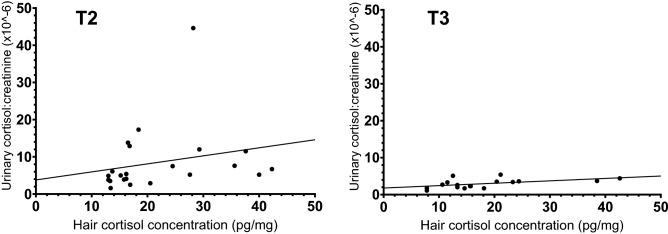
Correlation between hair cortisol concentration (pg/mg) and urinary cortisol:creatinine (× 10^-6) in shelter dogs. For sample collection moments T2 (n = 21) and T3 (n = 14).

A significant but moderate positive correlation between UCCR and HCC in shelter dogs was found on both T2 and T3 (Spearman’s Rho T2 ρ = 0.52, *p* = 0.01; T3 ρ = 0.55, *p* = 0.024, Fig. 3).

## Discussion

In this study we compared HCC in shelter dogs over different chronological time points during sheltering and after adoption with a shave/re-shave method, aiming to further validate HCC to measure chronic stress in dogs. We also compared pre-shelter levels with those of control pet dogs and explored the relation between HCC and urine cortisol levels in shelter dogs. In short, we found HCC levels of shelter dogs to be higher in samples of hair that grew during the first 6 weeks in the shelter than in samples collected at intake in the shelter, and to be higher than in two samples taken 6 weeks and 6 months after adoption into a new home. This implies that cortisol levels in the shelter were higher and thus dogs appear more stressed during the first shelter period. HCC levels from the intake samples of the shelter dogs did not differ from the control group of pet dogs, reflecting no overall difference in cortisol levels between the two groups when living in familiar homes. HCC was moderately but significantly correlated with mean urine cortisol creatinine ratio, showing a relation between the two measures.

Half of the dogs (27/52) were already adopted out or returned to the former owner after 6 weeks in the shelter and were therefore not available for hair sampling 6 weeks in the shelter. Dogs could still be in the shelter after 6 weeks for many reasons; breed type, body size and in-kennel behaviour are known to influence adoption likelihood^35^. Therefore, a bias can be present in the type of dogs that provided more samples at T2. For example, at T2 more large dogs provided samples than smaller dogs (> 30 kg 35% versus < 10 kg 17% of total dogs at T2, compared to 21% versus 35% in total). Another possibility is that dogs that cope worse with the shelter environment were still in the shelter at T2. However, as seen in Fig. 2B, these were not the dogs with higher HCC levels in general at T1, making this option less plausible.

HCC levels of dogs did not appear to be elevated during the period before the dogs were admitted to the shelter compared to after adoption, which implies no profound stressful pre-shelter period. However, it is possible that part of the hair shaft re-shaved at T2 did develop prior to the first shave at intake, as hair in the follicle and up to ~ 1 mm out of the follicle could not be shaved due to proximity to the skin. During the late anagen phase of hair follicles, hair follicles in dogs reach a length of ~ 2.5 mm^36^. Hair grows at a mean of 1 cm per month in humans^37^ and ranged from 5.3–12.0 mm per month in pigs and 3.5–17.0 mm per month in cattle^38^. In dogs, hair regrowth was a mean of 82% at 6–12 weeks after shaving in German Shepherds and Labrador Retrievers^17^ and fully regrown to pre-shave length in an average of 14.7 weeks in Labrador Retrievers^39^. Therefore, our re-shaved hair shaft 6 weeks in the shelter could contain ~ 3.5 mm of hair that had already developed up to weeks before intake at the shelter. To control for this ‘old’ hair growth, future studies can remove the first few millimetres of the hair shaft that are not of interest for analysis.

We found several dog-related factors to influence HCC. Interestingly, we found an effect of weight class, with smaller dogs having higher HCC than larger dogs. Urinary cortisol:creatinine levels were also higher in small shelter dogs than larger dogs in earlier studies^30,40–42^, which could be explained by relatively little creatinine production in smaller dogs as creatinine production is proportional to muscle mass^43^, however not relevant to HCC levels. Also, salivary cortisol levels were lower in giant/large dogs^44^ and were negatively correlated with body weight^41^, therefore cortisol levels in general seem to be higher in smaller dogs. Bowland et al.^27^ also found HCC to be negatively associated with size (based on morphometric measurements) in hunting village dogs in a reserve in Nicaragua, although this effect disappeared in a model with all factors incorporated. To our best knowledge, no other studies have investigated the effect of body size on HCC in dogs. However, it has been suggested that plasma cortisol varies with mass-specific metabolic rate in mammals, where larger mammals have lower cortisol levels^45^. Furthermore, an effect of sex on HCC was also found, with females having higher HCC than males. Higher HCC in the hair of female dogs has been found in recent studies in herding dogs^25^, solitary hunting breeds^26^, and hunting village dogs^27^, although reproductive status of the female (pregnancy or lactation) could have affected HCC. Other studies, however, did not find an effect of sex on HCC in dogs^21,24^. In general, across species, an inconsistent influence of sex on HCC has been found between studies^9^. However, female dogs showed a higher behavioural and HPA-axis response to stressors^1,46,47^ and it is known that HPA-axis responses are higher in females in rodents and humans as a result of circulating estradiol levels^48,49^, which can be an explanation for the higher HCC. We also found a puzzling interaction effect of age, where dogs of 5–7 year had higher HCC levels at T2 but lower at T3 and T4 than younger dogs of 1–4 year. However, the number of samples of dogs of 5–7 years was very low, and therefore results need to be interpreted with caution. No effect of age on HCC has been found before in dogs^21,25^ but for several species an age-dependent decline in HCC from young to adult age groups was found^9^. And last, stray dogs overall had lower HCC than relinquished dogs, but this difference was not visible in the intake samples in the shelter. Interestingly, Willen et al.^50^ also found no difference in HCC between relinquished and stray dogs at intake in a shelter. Therefore, it seems that stray dogs respond different to sheltering than relinquished dogs, which can therefore depend on previous life experiences. This supports the hypothesis that prior life history of dogs can mediate coping to a shelter environment^51^, although it must be mentioned that stray dogs in the Netherlands are mostly on the streets only for a few hours to a few days before they are captured and admitted to a shelter. To summarize, these dog-related factors need to be considered in future studies as their effect on HCC is present or not yet clear.

We found no overall effect of melanin type on HCC, but an effect that differed per sample collection moment. However, sample sizes per sample collection moment were often very low and therefore these results need to be interpreted with caution. Compared to eumelanin (brown-grey-black) hair samples, pheomelanin (blonde-yellow–red) contained lower HCC at T2, white contained higher HCC at T3, and mixed samples of hair contained lower HCC at T4. Therefore, we did not find a consistent effect of colour on HCC. So far, in the literature, the effect of colour on HCC is also inconsistent across and within species^9,52^. In general, due to the variety in hair colours between and within dogs, visual evaluation of the dominant pigment type in hair samples can be difficult. Therefore, chemical analysis of eumelanin and pheomelanin in dog hair sample or analysis of pigment genes in fundamental studies can help to objectively evaluate differences in HCC in dog hair samples. For example, in humans, the effect of hair pigmentation on HCC was studied by evaluating hair pigmentation genes^53^.

Overall, HCC in shelter dogs is highly promising to provide information on cortisol responses retrospectively and in the long-term. HCC can be a ‘stable’ measure for evaluating these responses of dogs during their lives, for example to evaluate lifestyles and personality traits in dogs^23,25,26^. For adaptation to a novel environment however, an acute changing situation may not be reflected as the HCC in the hair shaft gets ‘averaged’ or ‘diluted’ during analysis^54,55^ and therefore other matrices such as urine are a better choice for evaluating acute cortisol levels. In our study, HCC correlated moderately but significantly with UCCR levels, showing a form of construct validity. Correlations between cortisol matrices with different time spans are complex, as some matrices such as blood or urine reflect a ‘snap shot’ in time while others integrate all these moments in time, such as hair and nails^54^. Therefore, even when averaging urinary cortisol over a prolonged period, it can be difficult to find a relationship between HCC and urinary cortisol levels (such as in humans^56,57^). To relate HCC with cortisol levels in another biological substrate, nail tissue is a more logical option^28,58^, however cortisol levels in the nails of dogs have not yet been validated. Furthermore, behavioural responses to stress provide important information to draw conclusions on physiological responses such as HCC^1^, for example on the valence of the stress response, i.e., positive or negative arousal.

The average levels of HCC in our study are comparable to other studies. However, comparisons of HCC levels between studies are difficult, as cortisol extraction from the hair might depend on the way the hair is processed. For example, powdering the hair can result in a 3.5 fold increase in cortisol measurement compared to when the hair is only chopped^52^. More standardized protocols for both the analysis of HCC and the collection of the hairs would improve comparability of studies in this field. Shave/re-shave methods are preferred when aiming to evaluate cortisol levels over a certain period, as this assures a known timeline of cortisol incorporation into the new hair and avoids including follicles in the sample^10^. Also, better insight of cortisol deposition in different types of dog fur, for example related to hair growth (breed dependent), can improve reliability of this measure to evaluate cortisol levels in dogs.

To conclude, hair cortisol concentration in shelter dogs increased after 6 weeks spent in the shelter and correlated moderately with UCCR levels. Therefore, to improve canine welfare, HCC analysis can be a reliable and feasible non-invasive method to evaluate cortisol levels in shelter dogs, especially when comparing HCC levels over longer periods within dogs. A shave/re-shave method is preferred when aiming to evaluate HCC levels during a specific period. However, we and others^9,23,24^ raise concerns on methodological issues and missing information on how cortisol is deposited in the hair shaft in a broad range of dog breeds with different hair types. Therefore, further studies are needed to shed light on these concerns.

## Supplementary Information

Supplementary Information.

## Data Availability

The datasets generated during and/or analysed during the current study are available from the corresponding author on reasonable request.
