# Supplementary material for: Evaluation of hair cortisol as an indicator of long-term stress responses in dogs in an animal shelter and after subsequent adoption
Source: Sci Rep. 2022 Apr 21;12:5117. doi: 10.1038/s41598-022-09140-w (PMC9023568; doi:10.1038/s41598-022-09140-w)
Supplement: Supplementary file 1 — Supplementary Information. [file 41598_2022_9140_MOESM1_ESM.pdf]

Evaluation of hair cortisol as an indicator of long-term stress responses in dogs in an animal shelter and after subsequent adoption

Janneke Elisabeth van der Laan [a], Claudia Maureen Vinke [a], & Saskia Stefanie Arndt [a]
[a] Animal Behaviour group, Dept. Population Health Sciences – Div. Animals in Science and Society, Faculty of Veterinary Medicine, Utrecht University. P.O. Box 80166, 3584 CM Utrecht, The Netherlands.

Supplementary information

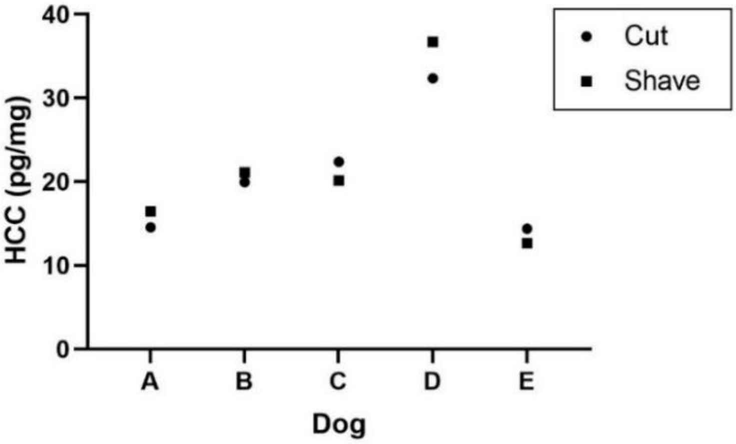

Supplementary Fig. 1. Pilot results of hair cortisol concentration (HCC) in cut versus shaved hair samples. Samples were taken from the same dogs (dog A-E) on the same location on the dog (neck region). No significant difference was found (two-tailed paired-samples t-test, t=-0.57, p=0.597).

Supplementary Table 1: Demographics per shelter dog (SD) and control pet dog (CPD). Estimated breed group [31], age class (in years, [30]), sex (female = f, male = m), neuter status (yes = y, no = n, unknown = ?), reason for admission to the shelter (relinquished = R, stray = S, crisis boarding = CB), body weight class (in kg), melanin type and the colour of the hair sample of the dogs.

Breed group	SD/ CPD	Age class	Sex	Neut. status	Reason adm.	Kennel history	Body weight	Melanin type	Colour hair samples
Ancient & spitz breeds	SD	1-4 yrs	f	?	R	?	10-20 kg	Mixed	Red, 30% black
Ancient & spitz breeds	SD	1-4 yrs	f	?	CB	?	<10 kg	Eumelanin	Brown
Ancient & spitz breeds	SD	1-4 yrs	m	n	S	?	<10 kg	White	White
Ancient & spitz breeds	SD	1-4 yrs	m	n	S	?	10-20 kg	Pheomelanin	Red Blonde
Ancient & spitz breeds	SD	1-4 yrs	m	y	R	?	10-20 kg	Eumelanin	Dark grey
Ancient & spitz breeds	SD	1-4 yrs	m	n	R	?	<10 kg	Mixed	Red Blonde, 30% black
Ancient & spitz breeds	SD	1-4 yrs	m	n	R	?	<10 kg	Mixed	White, 30% black
Herding dogs	SD	1-4 yrs	f	y	R	?	>30 kg	Mixed	White, few dark hairs
Herding dogs	SD	1-4 yrs	f	y	R	n	>20-30 kg	Agouti	Agouti
Herding dogs	SD	1-4 yrs	m	n	R	?	10-20 kg	Mixed	Red, 30% white, 10% black
Mastiff-like dogs	SD	1-4 yrs	f	n	R	?	>20-30 kg	Eumelanin	Blue
Mastiff-like dogs	SD	1-4 yrs	f	y	R	y	<10 kg	Eumelanin	Brown
Mastiff-like dogs	SD	1-4 yrs	f	n	S	?	>30 kg	Eumelanin	Black
Mastiff-like dogs	SD	1-4 yrs	f	n	R	n	>20-30 kg	Eumelanin	Light brown
Mastiff-like dogs	SD	1-4 yrs	m	y	R	?	>20-30 kg	Eumelanin	Brown
Mastiff-like dogs	SD	1-4 yrs	m	n	R	y	>20-30 kg	Eumelanin	Grey brown
Mastiff-like dogs	SD	1-4 yrs	m	n	S	?	>30 kg	Pheomelanin	Blonde
Mastiff-like dogs	SD	1-4 yrs	m	n	S	?	>30 kg	Eumelanin	Blue
Mastiff-like dogs	SD	1-4 yrs	m	n	R	?	>20-30 kg	Eumelanin	Brown
Mastiff-like dogs	SD	1-4 yrs	m	n	R	?	>30 kg	White	White
Mastiff-like dogs	SD	1-4 yrs	m	y	R	?	>30 kg	Eumelanin	Brown
Mastiff-like dogs	SD	1-4 yrs	m	n	S	?	10-20 kg	Eumelanin	Brown
Mastiff-like dogs	SD	1-4 yrs	m	n	S	?	>30 kg	Eumelanin	Brown
Mastiff-like dogs	SD	5-8 yrs	f	n	S	?	>20-30 kg	Eumelanin	Dark brown
Mastiff-like dogs	SD	5-8 yrs	m	n	S	?	>20-30 kg	Eumelanin	Dark brown
Mastiff-like dogs	SD	5-8 yrs	m	n	R	n	>20-30 kg	Pheomelanin	Blonde
Mastiff-like dogs	SD	9-13 yrs	m	n	S	?	10-20 kg	Eumelanin	Brown, 30% black

Mastiff-like dogs	SD	9-13 yrs	m	y	R	n	>30 kg	Eumelanin	Brown
Mixed (undefinable)	SD	?	m	n	CB	?	10-20 kg	Pheomelanin	Red Blonde
Mixed (undefinable)	SD	5-8 yrs	f	y	R	?	10-20 kg	Pheomelanin	Red
Mixed (undefinable)	SD	5-8 yrs	f	y	R	?	10-20 kg	Pheomelanin	Red Blonde
Mixed (undefinable)	SD	5-8 yrs	m	y	R	n	>20-30 kg	White	White
Retrievers	SD	1-4 yrs	f	?	S	?	>20-30 kg	Pheomelanin	Blonde
Retrievers	SD	1-4 yrs	f	n	S	?	>30 kg	Eumelanin	Black
Retrievers	SD	1-4 yrs	m	y	R	?	>30 kg	Eumelanin	Dark brown
Retrievers	SD	1-4 yrs	m	n	R	y	<10 kg	Pheomelanin	Dark red
Retrievers	SD	5-8 yrs	m	n	R	y	>30 kg	Eumelanin	Dark brown
Scent hounds	SD	9-13 yrs	m	y	R	y	<10 kg	Eumelanin	Black
Small terriers	SD	1-4 yrs	f	n	S	?	<10 kg	Pheomelanin	Red
Small terriers	SD	1-4 yrs	f	n	R	n	10-20 kg	Eumelanin	Black
Small terriers	SD	1-4 yrs	m	n	S	?	<10 kg	Mixed	Dark brown, 40% red
Small terriers	SD	1-4 yrs	m	y	R	n	<10 kg	Eumelanin	Brown
Small terriers	SD	1-4 yrs	m	y	R	?	<10 kg	Mixed	White, few red hairs
Small terriers	SD	1-4 yrs	m	y	S	?	<10 kg	Mixed	50% black 50% white
Small terriers	SD	5-8 yrs	f	n	R	n	10-20 kg	White	White
Small terriers	SD	5-8 yrs	m	y	R	?	<10 kg	Eumelanin	Black
Small terriers	SD	9-13 yrs	m	n	R	y	<10 kg	White	White
Spaniels	SD	1-4 yrs	m	y	R	y	10-20 kg	Pheomelanin	Red
Toy dogs	SD	1-4 yrs	f	y	R	?	<10 kg	Pheomelanin	Blonde
Toy dogs	SD	1-4 yrs	m	n	R	?	<10 kg	Pheomelanin	Blonde
Toy dogs	SD	9-13 yrs	m	n	S	?	<10 kg	Mixed	Red Blonde, 30% black
Working dogs	SD	1-4 yrs	f	n	CB	?	<10 kg	Pheomelanin	Blonde
Ancient & spitz breeds	CPD	1-4 yrs	f	y			<10 kg	Pheomelanin	Darkblonde/red
Ancient & spitz breeds	CPD	1-4 yrs	f	n			10-20 kg	White	White
Ancient & spitz breeds	CPD	1-4 yrs	m	y			<10 kg	Eumelanin	Light brown, 30% black
Ancient & spitz breeds	CPD	9-13 yrs	m	n			>20-30 kg	Mixed	White, 40% dark brown
Herding dogs	CPD	1-4 yrs	m	y			10-20 kg	Mixed	Brown, 40% white
Mastiff-like dogs	CPD	1-4 yrs	f	y			>20-30 kg	Eumelanin	Dark brown, 30% black
Mastiff-like dogs	CPD	1-4 yrs	f	y			>20-30 kg	Eumelanin	Dark brown
Mastiff-like dogs	CPD	1-4 yrs	m	y			>20-30 kg	Eumelanin	Black
Mastiff-like dogs	CPD	1-4 yrs	m	y			10-20 kg	Eumelanin	Black
Mastiff-like dogs	CPD	5-8 yrs	m	y			>20-30 kg	Eumelanin	Black
Mastiff-like dogs	CPD	5-8 yrs	m	y			>20-30 kg	White	White
Mixed (undefinable)	CPD	1-4 yrs	f	y			10-20 kg	Eumelanin	Brown, few black hairs
Mixed (undefinable)	CPD	1-4 yrs	m	y			10-20 kg	Eumelanin	Brown, 30% light brown
Retrievers	CPD	1-4 yrs	f	y			>20-30 kg	Eumelanin	Black
Retrievers	CPD	5-8 yrs	f	y			>20-30 kg	White	White
Scent hounds	CPD	1-4 yrs	m	n			<10 kg	Eumelanin	Black
Small terriers	CPD	1-4 yrs	m	y			10-20 kg	Pheomelanin	Red Blonde
Small terriers	CPD	1-4 yrs	m	y			<10 kg	Mixed	White, 10% black
Small terriers	CPD	5-8 yrs	f	y			<10 kg	Mixed	White, 30% brown
Toy dogs	CPD	1-4 yrs	m	y			<10 kg	Eumelanin	Brown, 20% dark brown
